# *QuickStats*: Age-Adjusted Suicide Rates,*^,^^^† ^^by Race/Ethnicity — National Vital Statistics System, United States, 2015–2016

**DOI:** 10.15585/mmwr.mm6714a6

**Published:** 2018-04-13

**Figure:**
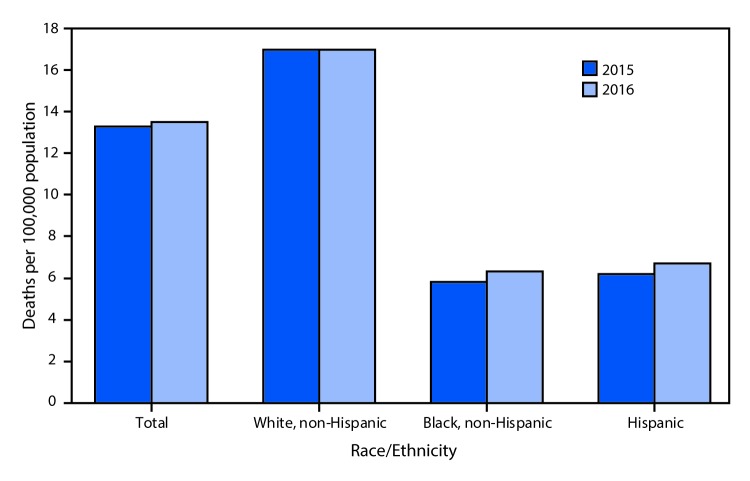
From 2015 to 2016, the age-adjusted suicide rate for the total U.S. population increased from 13.3 per 100,000 standard population to 13.5 (an increase of 1.5%). The rate increased from 5.8 to 6.3 (8.6%) for non-Hispanic blacks and from 6.2 to 6.7 (8.1%) for Hispanics; it remained unchanged for non-Hispanic whites. In both 2015 and 2016, the non-Hispanic white rate was nearly three times the non-Hispanic black rate and 2.5 times the rate for the Hispanic population.

**Source: **National Vital Statistics System. Underlying cause of death data, 1999–2016. https://wonder.cdc.gov/ucd-icd10.html.

For more information on this topic, CDC recommends the following link: https://www.cdc.gov/violenceprevention/suicide/index.html.

